# Supplementary material for: Maternal DNMT3A-dependent de novo methylation of the paternal genome inhibits gene expression in the early embryo
Source: Nat Commun. 2020 Oct 27;11:5417. doi: 10.1038/s41467-020-19279-7 (PMC7591512; doi:10.1038/s41467-020-19279-7)
Supplement: Supplementary file 1 — Supplementary Information [file 41467_2020_19279_MOESM1_ESM.pdf]

Supplementary Information for:

Richard Albert et al. Maternal DNMT3A-dependent *de novo* methylation of the paternal genome inhibits gene expression in the early embryo.

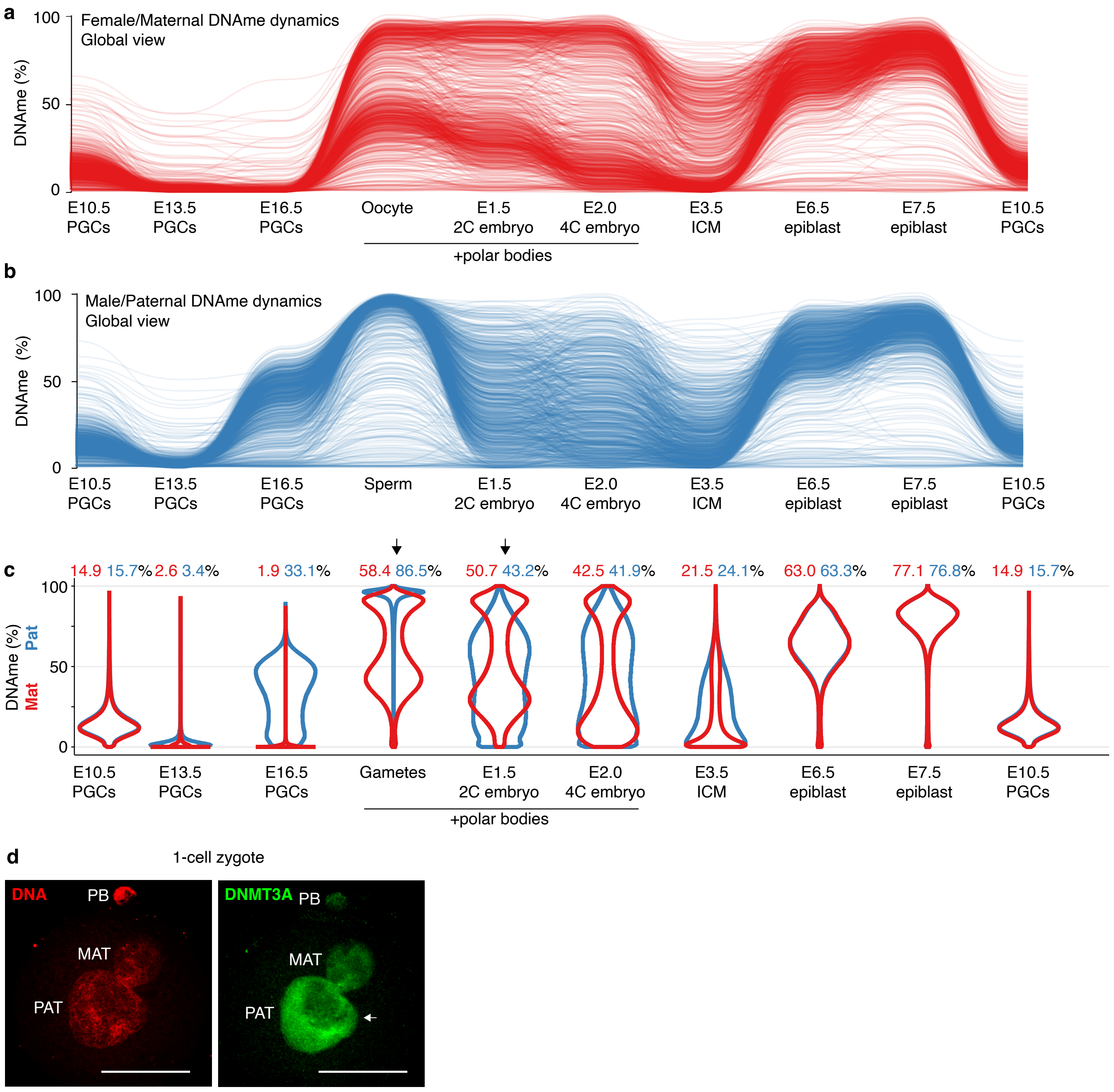

Supplementary Figure 1. Female/maternal and male/paternal DNAme level dynamics during gametogenesis and embryonic development. a-b Parallel coordinate plots illustrating average DNAme levels over 2 kb genomic bins during **a** female/maternal and **b** male/paternal gametogenesis and embryonic development. Allele-specific analysis of WGBS data yielded 176,240 bins (roughly 15% of the mouse genome) overlapping at least 5 informative CpGs for which we could infer both maternal and paternal DNAme levels in all datasets. A random set of 1,000 bins on chromosome 19 are shown. MII oocyte, 2C and 4C embryo datasets include polar bodies. Note the methylated loci on the maternal allele of preimplantation embryos (2C to ICM stage) correspond to actively transcribed regions in the oocyte (see[1]). **c** Distribution of global DNAme levels during gametogenesis and embryonic development. Mean DNAme percentages for each stage (red: female/maternal, blue: male/paternal) are shown above each violin plot. Arrows indicate the stages used to measure DNAme level change pre- and post-fertilization. **d** IF analysis of DNMT3A in a representative 1 cell zygote. Zygotic DNA is counterstained with propidium iodide (PI). Scale bar: 50 µm PB; polar body. The white arrow indicates DNMT3A straining in the paternal pronucleus. Experiment was conducted 5 times. Source data are provided as a Source Data file.

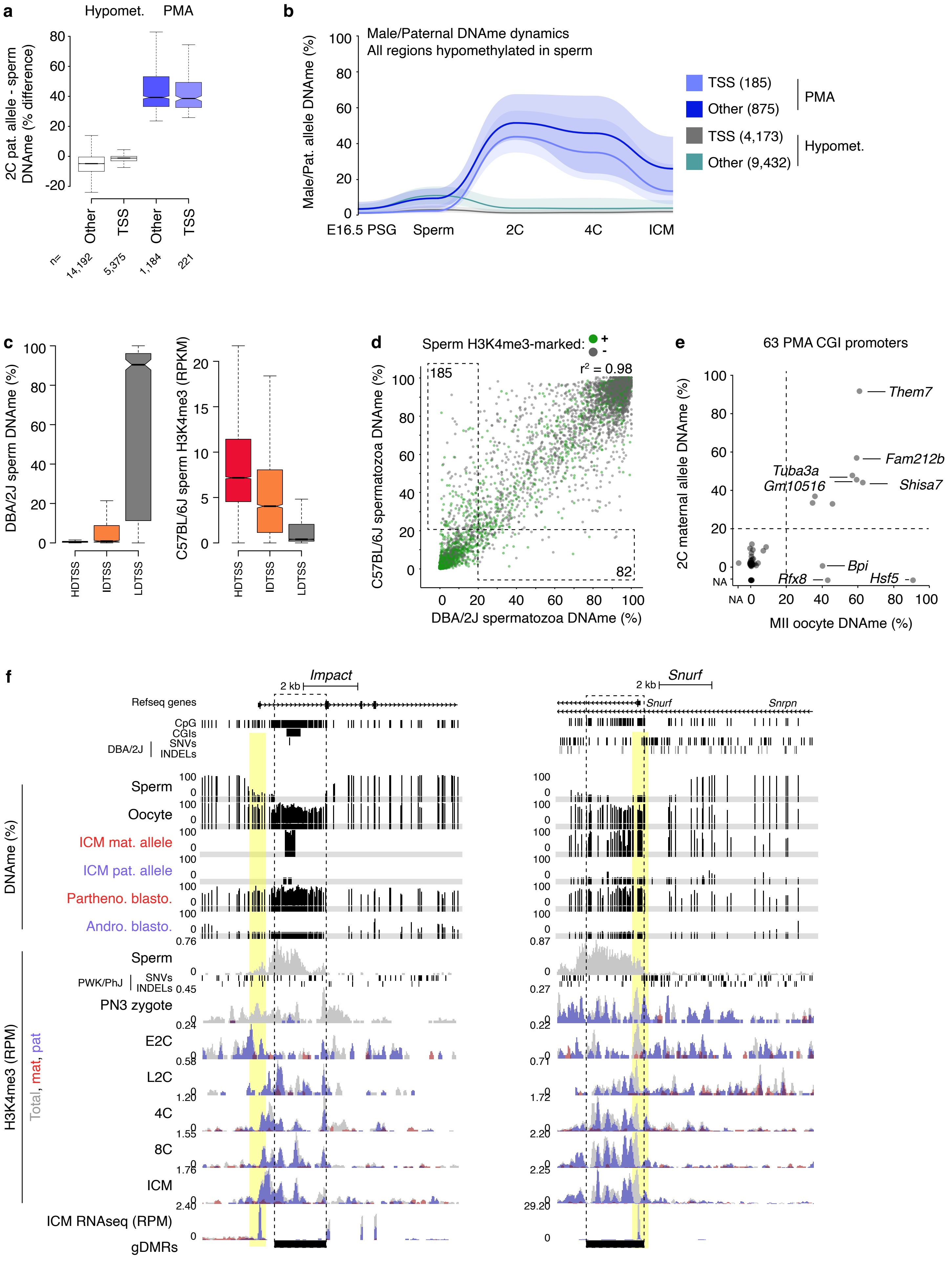
a
Hypomet.
PMA
2C pat. allele - sperm DNAme (% difference)
80
60
40
20
0
-20
Other
TSS
Other
TSS
n=
14,192
5,375
1,184
221
b
Male/Paternal DNAme dynamics
All regions hypomethylated in sperm
Male/Pat. allele DNAme (%)
100
80
60
40
20
0
E16.5 PSG
Sperm
2C
4C
ICM
TSS (185)
Other (875)
PMA
TSS (4,173)
Other (9,432)
Hypomet.
c
DBA/2J sperm DNAme (%)
C57BL/6J sperm H3K4me3 (RPKM)
HDTSS
IDTSS
LDTSS
d
Sperm H3K4me3-marked:
+
-
185
r^2 = 0.98
82
C57BL/6J spermatozoa DNAme (%)
DBA/2J spermatozoa DNAme (%)
e
63 PMA CGI promoters
2C maternal allele DNAme (%)
MII oocyte DNAme (%)
NA
Them7
Fam212b
Tuba3a
Gm10516
Shisa7
Bpi
Rfx8
Hsf5
f
Impact
Snurf
2 kb
Refseq genes
Snrpn
CpG
CGIs
SNVs
INDELs
DBA/2J
DNAme (%)
Sperm
Oocyte
ICM mat. allele
ICM pat. allele
Partheno. blasto.
Andro. blasto.
H3K4me3 (RPM)
Total, mat, pat
PWK/PhJ
PN3 zygote
E2C
L2C
4C
8C
ICM
ICM RNAseq (RPM)
gDMRs

Supplementary Figure 2. Defining hypomethylated CGI promoters in sperm. a Boxplot showing the distribution of DNAme differences between the paternal allele in 2C embryos and mature sperm over autosomal regions that remain hypomethylated (<20% DNAme, "hypomet." n=19,567) or those that show paternal DNA methylation acquisition (defined as a ≥30% gain, "PMA" n=1,405). Regions shown are subcategorized as "TSS" if they overlap a RefSeq-annotated genic TSS (+/- 300 bp) or "Other" if not. Boxplots show the median (line inside the box), where 50% of the data are distributed (the box), and whiskers denote the values lying within 1.5 times the interquartile range. **b** Parallel coordinate plot showing male/paternal DNAme level dynamics in sperm and the preimplantation embryo over regions defined in a for which sufficient data was available in each dataset. The number of reported regions for each category is indicated on the right. The median (bold line) and 25 and 75th quartiles (shaded area) are shown. **c** Autosomal TSSs were classified based on CpG density as described previously[2], and the distribution of DNAme and H3K4me3 levels for each promoter category is shown. HD: high CpG density (n=9,931), ID: intermediate CpG density (n=3,411), LD: low CpG density (n=9,243). Boxplots elements as defined in a. **d** 2D scatterplot illustrating the correlation of DNAme levels between C57BL/6J and DBA/2J sperm. Data points are coloured by H3K4me3 enrichment in C57BL/6J sperm. Only promoters for which we calculated DNAme levels in both datasets (at least 2 CpGs with 5X coverage separated by >1 sequencing read length) are shown (n=20,583). 20,163 promoters had consistent DNAme levels (difference <20%) between strains. **e** Maternal allele DNAme levels over CGI promoters that show PMA (both datasets include polar bodies). NA: no data available for that stage. **f** UCSC genome browser screenshots of the *Impact* and *Snurf* paternally expressed imprinted genes. Promoters are highlighted in yellow, gametic DMRs by a dashed box and the location of informative CpGs (5X coverage) for each WGBS dataset are highlighted in grey. Embryonic H3K4me3 and ICM RNA-seq data are represented as a composite track containing total (allele-agnostic, grey), maternal (red) and paternal (blue) genomic tracks. NCBI Refseq genes, all CpG dinucleotides, CpG islands and genetic variants used in our allele-specific analyses (SNVs and INDELs) are also included. Mat.: maternal, Pat.: paternal, Partheno.: parthenogenetic, Andro.: androgenetic, Blasto.: blastocyst, PN3: pronuclear stage 3, E2C: early 2 cell, L2C: late 2 cell, 4C: 4 cell, 8C: 8 cell, ICM: inner cell mass cells. Source data are provided as a Source Data file.

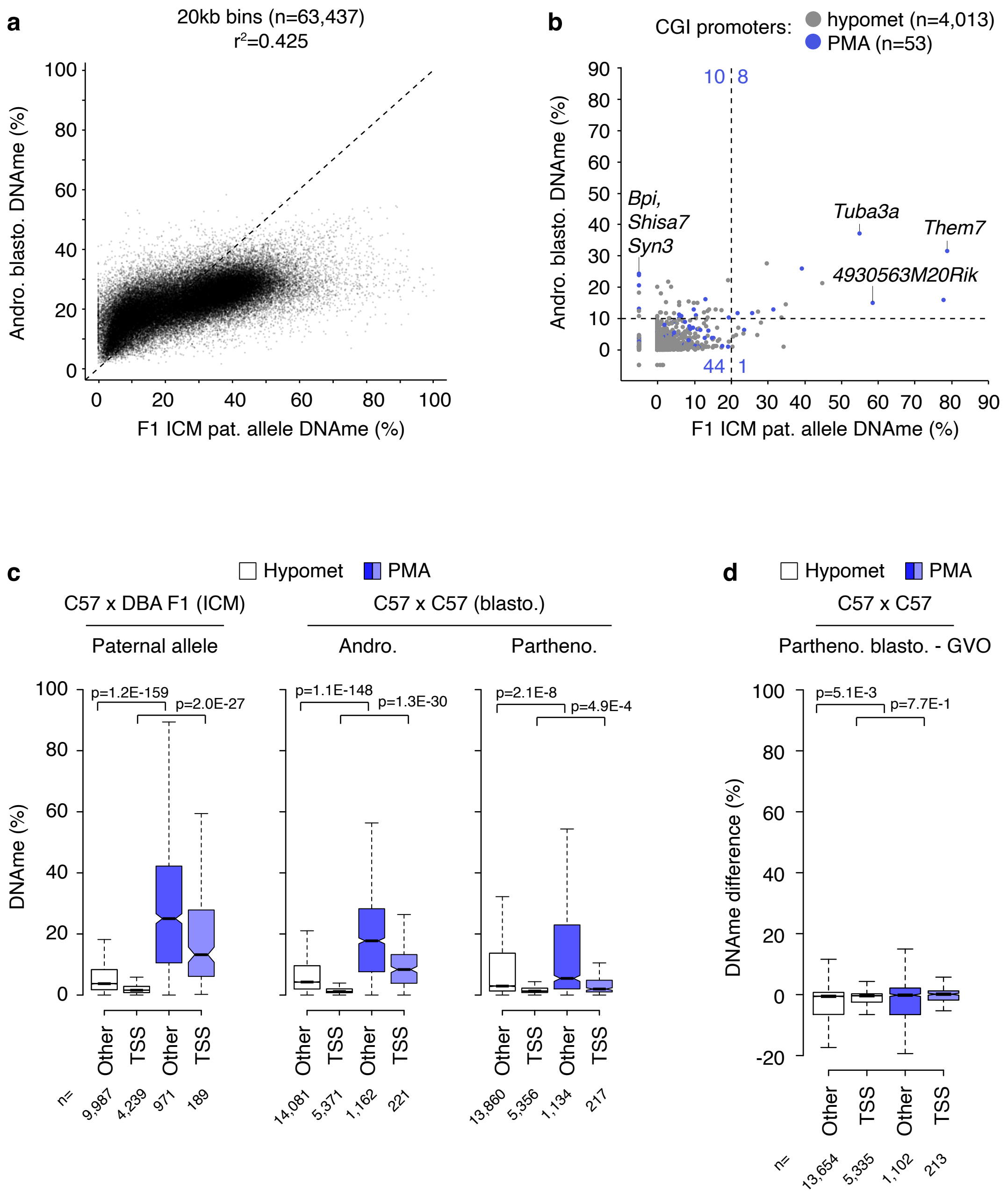

Supplementary Figure 3. WGBS analysis of uniparental E3.5 blastocysts. a Scatterplot showing genome-wide differences in DNAme levels over 20kb bins between the paternal allele of E3.5 C57 x DBA F1 hybrid ICM cells and androgenetic (C57 x C57) blastocysts. 20kb bins overlapping >3 informative CpGs (covered by 5X reads and separated by >1 sequencing read length) in both datasets are reported (n=63,437). **b** Scatterplot showing the relationship between CGI promoter DNAme levels on the paternal allele of normal F1 hybrid ICM cells and androgenetic blastocysts. Of note, the mean DNAme level (% +/- stdev) of the paternal allele over all autosomal CGI promoters is 1.5 (+/- 2.6%) and 1.7 (+/- 1.1%) in in F1 hybrid ICM and androgenotes, respectively. **c** Boxplots showing the distribution of DNAme levels over all TSS or “Other” regions (defined as in Fig. S2a) that show PMA in E3.5 F1 hybrid ICM, androgenetic blastocysts and parthenogenetic blastocyst. The number of loci represented is indicated below each plot. Boxplots show the median (line inside the box), where 50% of the data are distributed (the box), and whiskers denote the values lying within 1.5 times the interquartile range. Outliers not shown. Two-sided t-tests assuming unequal variances were performed.
d Boxplots showing the distribution of the differences in DNAme level between parthenogenetic blastocysts and germinal vesicle oocytes (GVOs, data does not contain polar bodies). Note that the intermediate DNAme levels in parthenogenetic blastocysts mirror those of the oocyte and therefore are unlikely to reflect de novo methylation following fertilization. The number of loci represented is indicated below each plot. Boxplots elements as defined in c. Two-sided t-tests assuming unequal variances were performed. Source data are provided as Source Data and Supplementary Data 4 files.

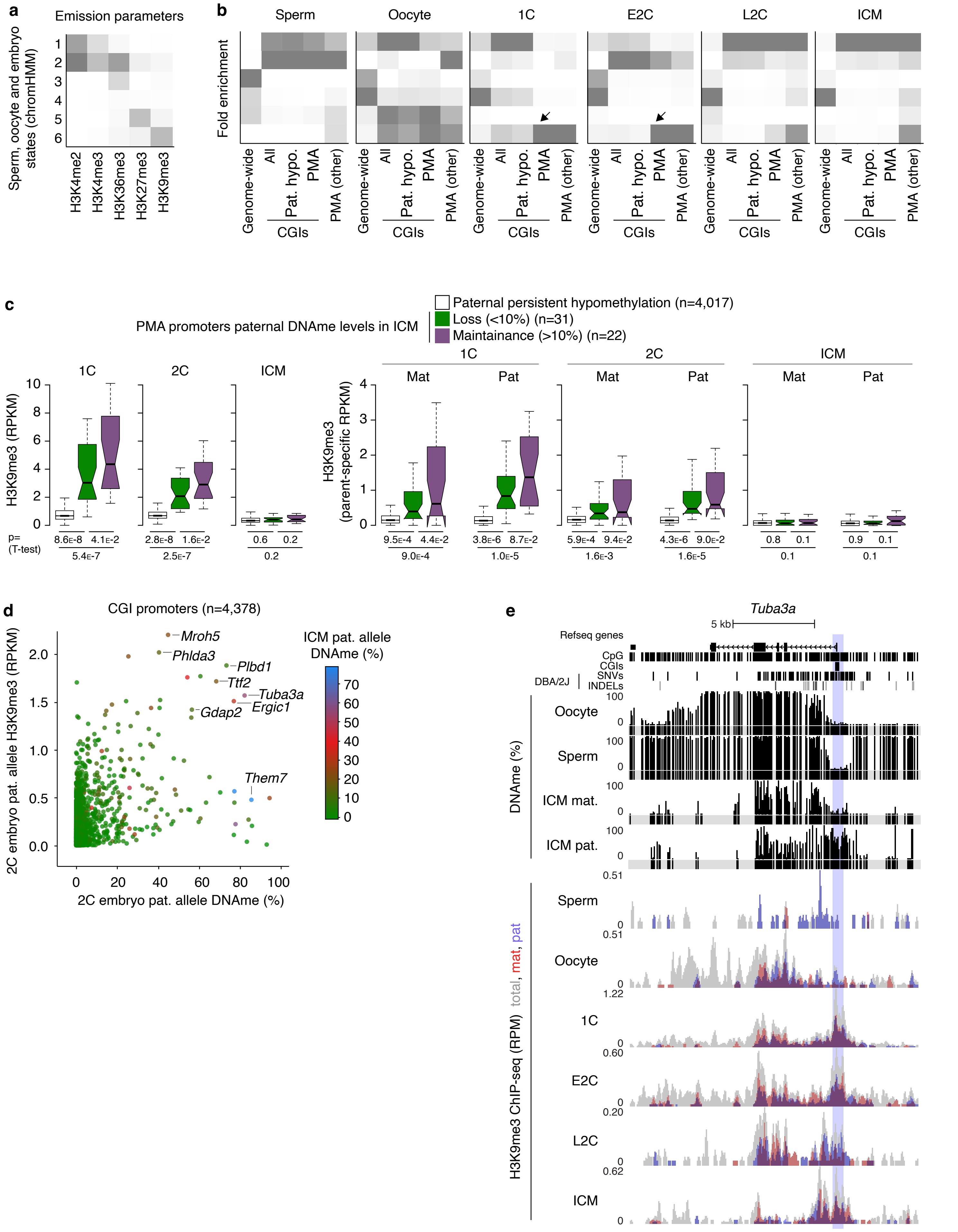

a
Emission parameters
Sperm, oocyte and embryo states (chromHMM)
1
2
3
4
5
6
H3K4me2
H3K4me3
H3K36me3
H3K27me3
H3K9me3
b
Sperm
Oocyte
1C
E2C
L2C
ICM
Fold enrichment
Genome-wide
All
Pat. hypo.
PMA
PMA (other)
CGIs
c
PMA promoters paternal DNAme levels in ICM
Paternal persistent hypomethylation (n=4,017)
Loss (<10%) (n=31)
Maintainance (>10%) (n=22)
1C
2C
ICM
H3K9me3 (RPKM)
H3K9me3 (parent-specific RPKM)
Mat
Pat
p= (T-test)
8.6E-8
4.1E-2
5.4E-7
2.8E-8
1.6E-2
2.5E-7
0.6
0.2
0.2
9.5E-4
4.4E-2
9.0E-4
3.8E-6
8.7E-2
1.0E-5
5.9E-4
9.4E-2
1.6E-3
4.3E-6
9.0E-2
1.6E-5
0.8
0.1
0.1
0.9
0.1
0.1
d
CGI promoters (n=4,378)
2C embryo pat. allele H3K9me3 (RPKM)
2C embryo pat. allele DNAme (%)
ICM pat. allele DNAme (%)
Mroh5
Phlda3
Plbd1
Ttf2
Tuba3a
Ergic1
Gdap2
Them7
e
Tuba3a
5 kb
Refseq genes
CpG
CGIs
SNVs
INDELs
DBA/2J
DNAme (%)
Oocyte
Sperm
ICM mat.
ICM pat.
H3K9me3 ChIP-seq (RPM) total, mat, pat
Sperm
Oocyte
1C
E2C
L2C
ICM

Supplementary Figure 4. Relationship between histone PTMs and PMA. a Six distinct chromatin states were identified genome-wide using ChromHMM and ChIP-seq data (H3K4me2, H3K4me3, H3K36me3, H3K27me3 and H3K9me3) from spermatozoa, MII oocytes, 1C, early 2C, late 2C and blastocyst-stage embryos. The probability that a histone modification is found within each state is shown. **b** The relative enrichment of each chromatin state genome-wide and over genomic regions of interest, including all autosomal CGI promoters (n=13,342), those that show persistent paternal DNA hypomethylation following fertilization (n=4,315), CGI promoters that show PMA (n=63), and regions exclusive of a TSS that show PMA (n=1,307) are shown. **c** The distribution of total (left), and parent allele-specific H3K9me3 (right) levels over CGI promoters in 1C, E2C and blastocyst-stage embryos is shown. CGI promoters were categorized by paternal DNAme dynamics, including persistent hypomethylation following fertilization (n=4,017), PMA genes showing loss of paternal DNAme by the blastocyst stage (n=31), and PMA genes showing persistence of paternal DNAme in the blastocyst (n=22). Boxplots show the median (line inside the box), where 50% of the data are distributed (the box), and whiskers denote the values lying within 1.5 times the interquartile range. Two-sided t-tests assuming unequal variances were performed. **d** Scatterplot illustrating the association between H3K9me3 and DNAme levels on the paternal allele at the 2C stage and maintenance of DNAme on the paternal allele in ICM cells. **e** UCSC genome browser screenshot of the *Tuba3a* locus illustrating DNAme levels in gametes and the ICM as well as maternal (red) and paternal (blue) H3K9me3 levels. The CpG rich promoter of *Tuba3a* is highlighted in blue and the location of informative CpGs (5X coverage) for each WGBS dataset are highlighted in grey. The genomic locations for NCBI Refseq genes, all CpG dinucleotides, CpG islands and genetic variants used in our allele-specific analyses (SNVs and INDELs) are also shown. H3K9me3 ChIP-seq data are represented as composite tracks containing total (allele-agnostic, grey), maternal (red) and paternal (blue) genomic tracks. Source data are provided as a Source Data file.

a

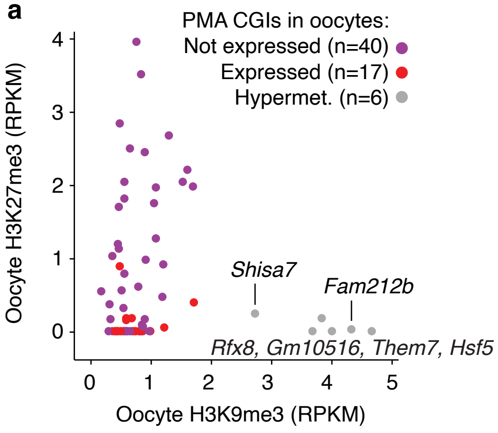

Supplementary Figure 5. Transcriptionally silenced PMA CGI promoters in oocytes are either DNA hypermethylated and H3K9me3-marked or hypomethylated and H3K27me3-marked in oocytes and the early embryo. a Scatterplot showing H3K9me3 and K3K27me3 enrichment in MII oocytes. Data points are coloured as in Fig. 3b. **b** UCSC genome browser screenshots of the transcriptionally silenced *Shisa7* and *Fam212b* loci illustrating hypermethylation and H3K9me3-enrichment of their CGI promoters in oocytes. Allele-specific H3K27me3, H3K9me3 and expression data from oocytes and the early embryo are included. 1C: 1 cell zygote, E2C: early 2 cell, L2C: late 2 cell, ICM: inner cell mass cells. Source data are provided as a Source Data file.

b

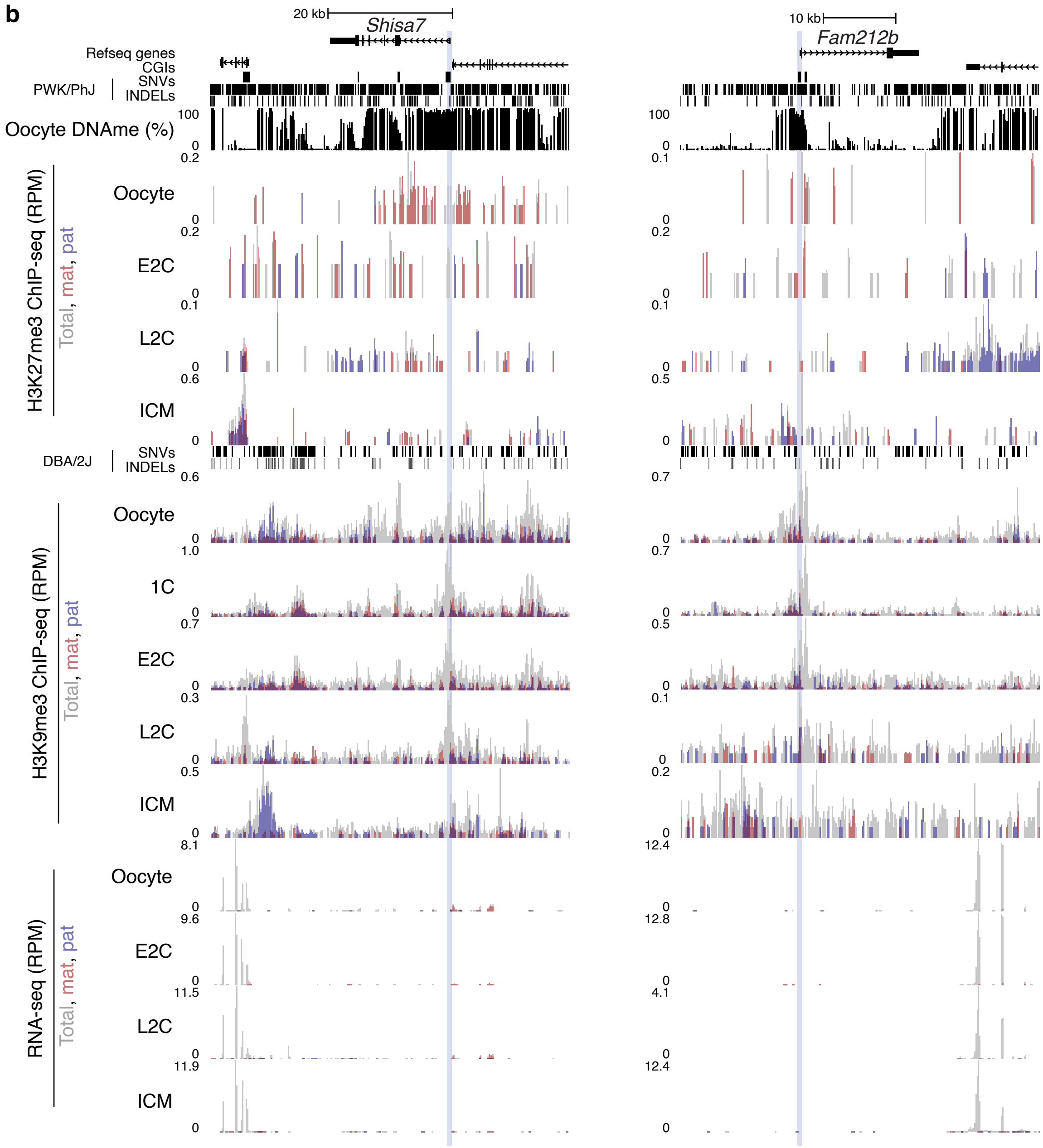

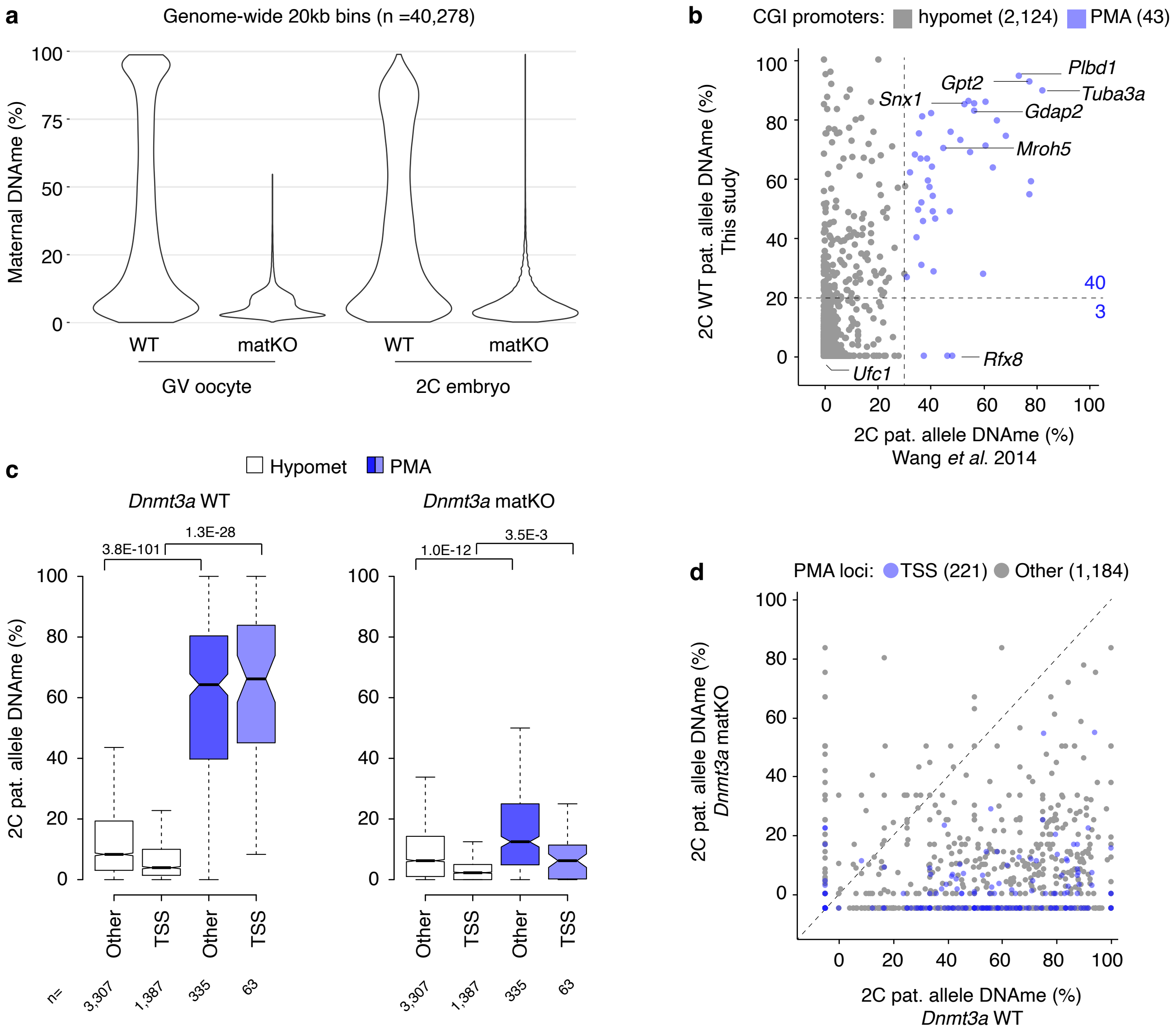

Supplementary Figure 6. DNAme analysis of maternal *Dnmt3a* KO embryos. a Distribution of maternal-allele DNAme levels over 20kbp genomic bins in WT and *Dnmt3a* matKO GV oocytes and 2 cell embryos. 20kb bins overlapping >3 informative CpGs (covered by 5X reads and separated by >1 sequencing read length) in all 4 datasets are reported (n=40,278). GV oocyte data from Shirane et al. 2013[3]. **b** Paternal allele CGI promoter DNAme level association between wild-type 2C data generated in this study and those from Wang et al. 2014[4]. Genes are coloured by paternal DNAme dynamics immediately following fertilization: grey; persistent hypomethylation on the paternal genome (paternal hypomet, n=2,124), blue; paternal DNAme acquisition (PMA, n=43). Paternal DNAme levels over CGI promoters were reported if they were covered by at least 5 informative CpGs (covered by 1X read and separated by >1 sequencing read length) in our 2C WGBS datasets. **c** Boxplots illustrating the distribution of paternal allele DNAme levels in WT and *Dnmt3a* matKO 2C embryos over all regions that show persistent hypomethylation (n=4,694) or PMA (n=398). TSS or "Other" regions are defined as in Fig. S2a and regions overlapping at least 5 allele-specific CpGs (covered by 1X read and separated by >1 sequencing read length) in both datasets are shown. Boxplots show the median (line inside the box), where 50% of the data are distributed (the box), and whiskers denote the values lying within 1.5 times the interquartile range. Outliers not shown. Two-sided t-tests assuming unequal variances were performed. **d** Scatterplot depicting paternal allele DNAme levels in WT and *Dnmt3a* matKO 2C embryos over all regions that show PMA. Data points with values <0 indicate insufficient information for calling DNAme levels in that particular dataset. Source data are provided as Source Data and Supplementary Data 5 files.

Richard_Albert_Supplementary_Figure_7

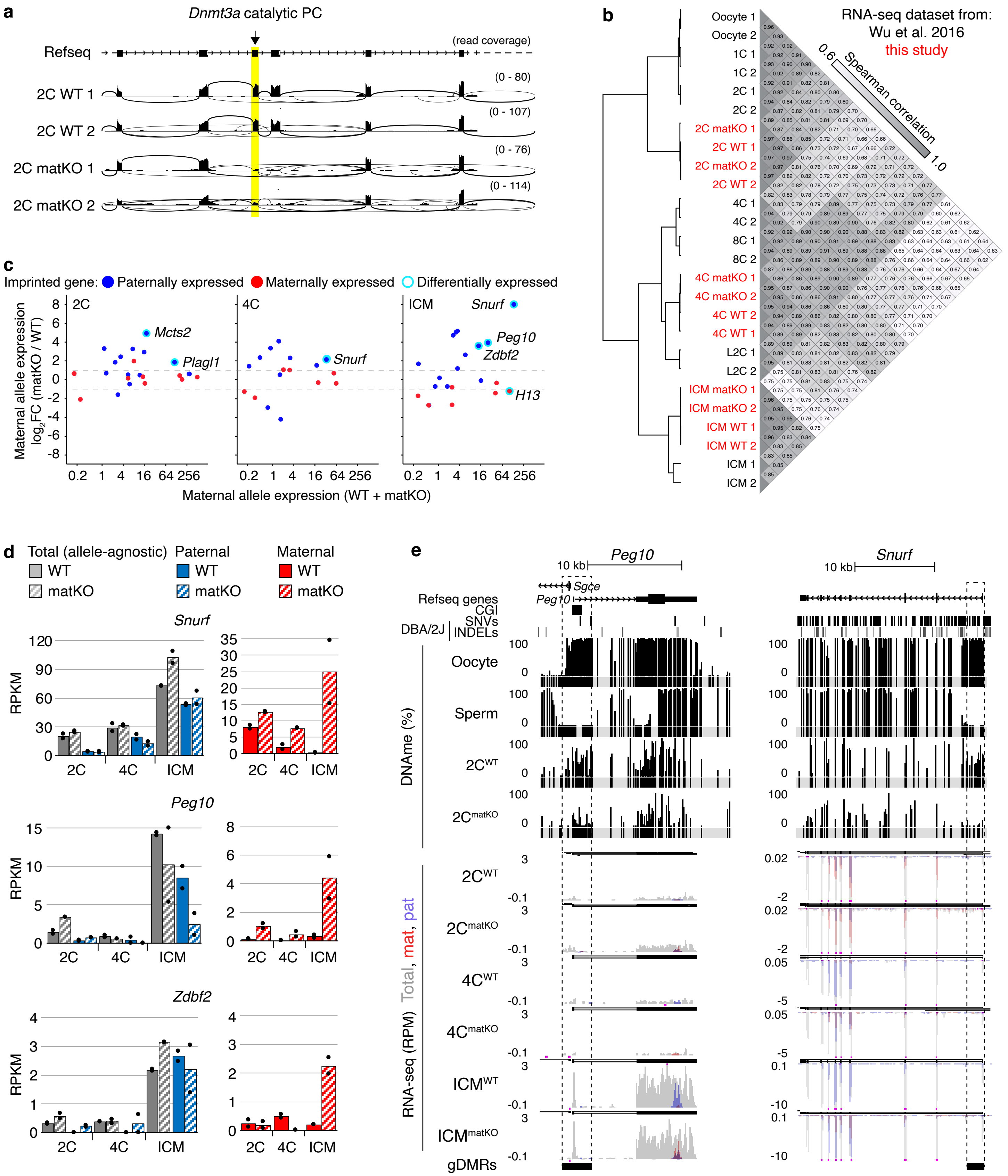

Supplementary Figure 7. Transcriptomic analysis of maternal *Dnmt3a* KO embryos. a 2 cell strand-specific RNA-seq sashimi plot illustrating deletion of the catalytic exon of *Dnmt3a*. **b** Spearman correlation of autosomal gene expression (log_{10}(RPKM+0.1) in datasets mined from Wu et al. 2016[5] (black) and generated in this study (red). **c** 2D scatterplots showing change in known imprinted gene expression from the maternal allele. Data points are coloured by whether they are normally paternally (blue) or maternally (red) expressed in somatic cells. Genes showing a change in expression of ≥2-fold are highlighted in teal. **d** Bar plots illustrating differential expression of select imprinted genes from both alleles (total, grey), the paternal genome (blue) and the maternal genome (red) in wild-type (filled bars) and *Dnmt3a* matKO (striped bars) embryos. Each bar represents the mean expression value of two biological replicates (dots) in RPKM. **e** UCSC genome browser screenshots of the paternally expressed imprinted genes *Peg10* and *Snurf* illustrating loss of imprinting. Gametic DMRs are indicated by a dashed box and the location of informative CpGs (5X coverage) for each WGBS dataset are highlighted in grey. Strand-specific RNA-seq data is represented as a composite track of biological duplicates containing total (allele-agnostic, grey), maternal (red) and paternal (blue) genomic tracks. De novo assembly of transcripts is included above each RNAseq dataset. NCBI Refseq genes, all CpG dinucleotides, CpG islands and genetic variants used in our allele-specific analyses (SNVs and INDELs) are also included. Source data are provided as Source Data and Supplementary Data 6 files.

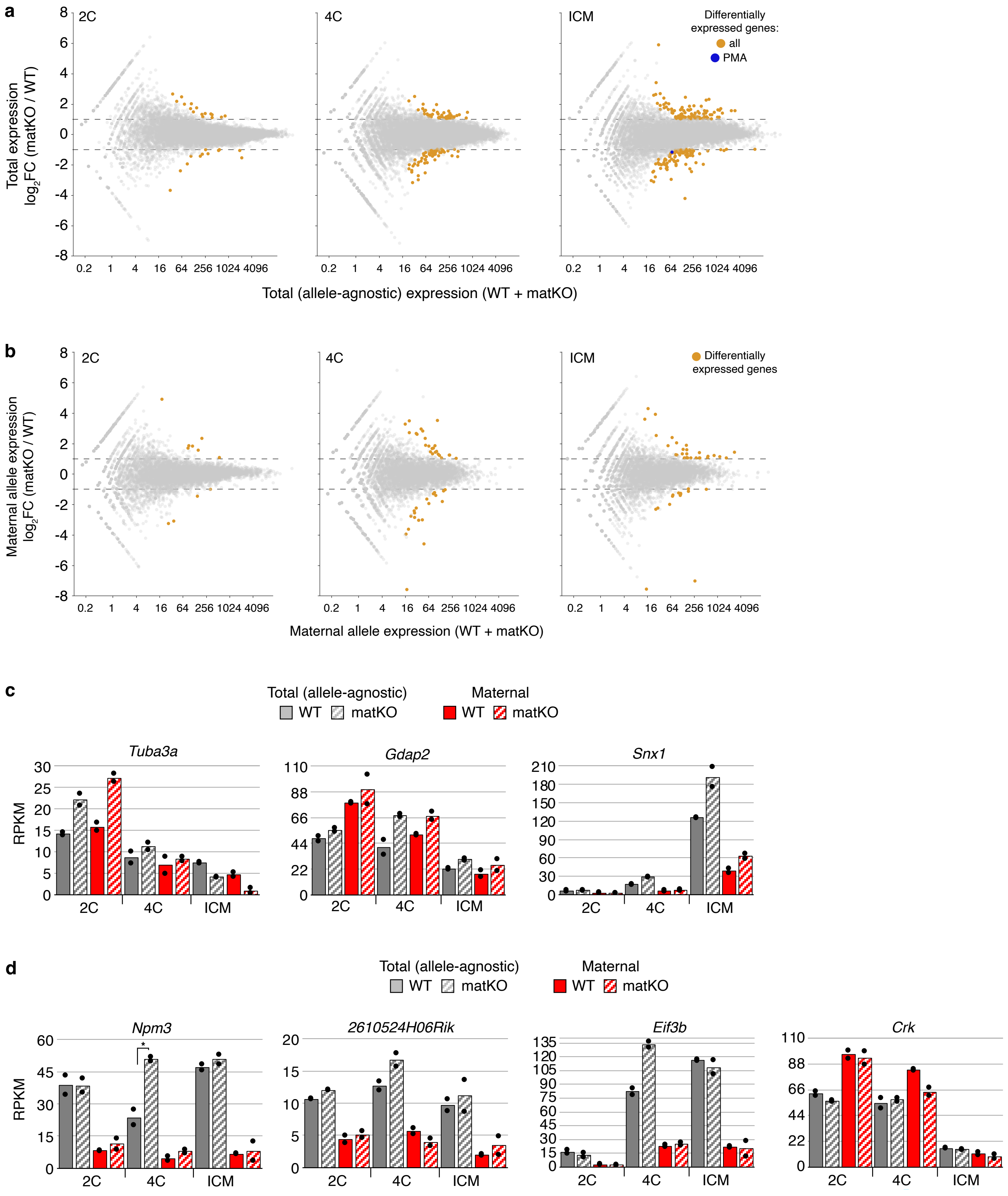
a
2C
4C
ICM
Differentially expressed genes:
all
PMA
Total expression
log_2FC (matKO / WT)
Total (allele-agnostic) expression (WT + matKO)
b
Differentially expressed genes
Maternal allele expression
log_2FC (matKO / WT)
Maternal allele expression (WT + matKO)
c
Total (allele-agnostic)
Maternal
WT
matKO
Tuba3a
Gdap2
Snx1
RPKM
d
Npm3
2610524H06Rik
Eif3b
Crk

Supplementary Figure 8. Allele-agnostic and maternal-allele analysis of the change in CGI promoter gene expression in *Dnmt3a* matKO embryos. a 2D scatterplots showing average (x-axis) and differential (y-axis) total (allele-agnostic) expression between wild-type (WT) and maternal knock-out (matKO) 2C, 4C embryos and ICM cells. Genes showing a change in expression of ≥2-fold are highlighted in orange, and those that show PMA are coloured in blue. **b** 2D scatterplots showing average (x-axis) and differential (y-axis) maternal-allele between WT and matKO 2C, 4C embryos and ICM cells. Legend as in **a**. No genes that show PMA at their CGI promoters were substantially upregulated from the maternal allele. **c-d** Bar charts illustrating differential expression of select genes from both alleles (grey) and the maternal genome (red) in wild-type (filled bars) and *Dnmt3a* matKO (striped bars) embryos. Each bar represents the mean expression value of two biological replicates (dots) in RPKM. Source data are provided as a Source Data file.

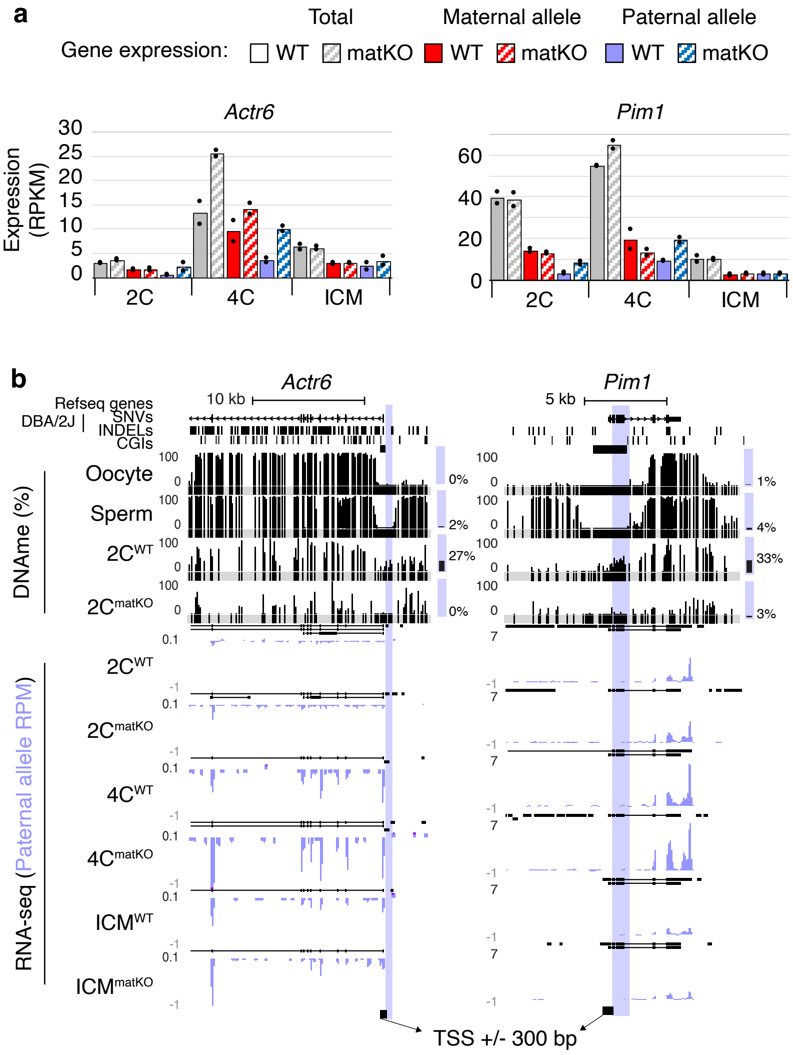

Supplementary Figure 9. *Actr6* and *Pim1* are upregulated from the paternal allele in *Dnmt3a* matKO 4C embryos and their TSSs are adjacent to regions that undergo PMA. a Bar charts illustrating differential expression of *Actr6* and *Pim1* from both alleles (grey), the maternal genome (red) and the paternal genome (blue) in wild-type (filled) and *Dnmt3a* matKO (striped) embryos. Each bar represents the mean expression value (in RPKM) of two biological replicates (dots). **b** UCSC genome browser screenshots of the *Actr6* and *Pim1* loci, as in Fig. 5. The TSS regions (+/- 300 bp) are shown at the bottom of each panel, and the regions undergoing PMA are highlighted in blue. Note that the latter do not overlap with the TSS regions. Source data are provided as a Source Data file.

Supplementary References

1 Brind'Amour, J. et al. LTR retrotransposons transcribed in oocytes drive species-specific and heritable changes in DNA methylation. Nature Communications 9, 3331 (2018).

2 Weber, M. et al. Distribution, silencing potential and evolutionary impact of promoter DNA methylation in the human genome. Nat Genet 39, 457–466 (2007).

3 Shirane, K. et al. Mouse oocyte methylomes at base resolution reveal genome-wide accumulation of non-CpG methylation and role of DNA methyltransferases. PLoS Genet 9, e1003439 (2013).

4 Wang, L. et al. Programming and inheritance of parental DNA methylomes in mammals. Cell 15, 979–991 (2014).

5 Wu, J. et al. The landscape of accessible chromatin in mammalian preimplantation embryos. Nature 534, 652–657 (2016).
