# Supplementary material for: Maternal DNMT3A-dependent de novo methylation of the paternal genome inhibits gene expression in the early embryo
Source: Nat Commun. 2020 Oct 27;11:5417. doi: 10.1038/s41467-020-19279-7 (PMC7591512; doi:10.1038/s41467-020-19279-7)
Supplement: Supplementary file 3 — Description of Additional Supplementary Files [file 41467_2020_19279_MOESM3_ESM.pdf]

Description of Additional Supplementary Files

File Name: Supplementary Data 1
Description: Processed data and lists of genes used to generate figures in this study.

File Name: Supplementary Data 2
Description: List of datasets mined and generated in this study.

File Name: Supplementary Data 3
Description: Genome-wide DNAme levels reported in Figure 1a.

File Name: Supplementary Data 4
Description: Genome-wide DNAme levels reported in Supplementary Figure 3a.

File Name: Supplementary Data 5
Description: Genome-wide DNAme levels reported in Supplementary Figure 6a.

File Name: Supplementary Data 6
Description: Gene expression data reported in Supplementary Figure 7b.
