# Supplementary material for: Maternal DNMT3A-dependent de novo methylation of the paternal genome inhibits gene expression in the early embryo
Source: Nat Commun. 2020 Oct 27;11:5417. doi: 10.1038/s41467-020-19279-7 (PMC7591512; doi:10.1038/s41467-020-19279-7)
Supplement: Supplementary file 10 — Reporting Summary [file 41467_2020_19279_MOESM10_ESM.pdf]

nature research

Corresponding author(s): Matthew Lorincz

Last updated by author(s): August 22 2020

# Reporting Summary

Nature Research wishes to improve the reproducibility of the work that we publish. This form provides structure for consistency and transparency in reporting. For further information on Nature Research policies, see Authors & Referees and the Editorial Policy Checklist .

## Statistics

For all statistical analyses, confirm that the following items are present in the figure legend, table legend, main text, or Methods section.

| n/a | Confirmed | |
|---|---|---|
| ☐ | ☒ | The exact sample size (*n*) for each experimental group/condition, given as a discrete number and unit of measurement |
| ☐ | ☒ | A statement on whether measurements were taken from distinct samples or whether the same sample was measured repeatedly |
| ☐ | ☒ | The statistical test(s) used AND whether they are one- or two-sided<br>*Only common tests should be described solely by name; describe more complex techniques in the Methods section.* |
| ☒ | ☐ | A description of all covariates tested |
| ☐ | ☒ | A description of any assumptions or corrections, such as tests of normality and adjustment for multiple comparisons |
| ☐ | ☒ | A full description of the statistical parameters including central tendency (e.g. means) or other basic estimates (e.g. regression coefficient) AND variation (e.g. standard deviation) or associated estimates of uncertainty (e.g. confidence intervals) |
| ☐ | ☒ | For null hypothesis testing, the test statistic (e.g. *F*, *t*, *r*) with confidence intervals, effect sizes, degrees of freedom and *P* value noted<br>*Give P values as exact values whenever suitable.* |
| ☒ | ☐ | For Bayesian analysis, information on the choice of priors and Markov chain Monte Carlo settings |
| ☒ | ☐ | For hierarchical and complex designs, identification of the appropriate level for tests and full reporting of outcomes |
| ☒ | ☐ | Estimates of effect sizes (e.g. Cohen's *d*, Pearson's *r*), indicating how they were calculated |

*Our web collection on statistics for biologists contains articles on many of the points above.*

## Software and code

Policy information about availability of computer code

| | |
|---|---|
| Data collection | Data was collected using standard Illumina software for the HiSeq 1500 and 2500 platforms. WGBS: HCS v2.2.68 and RTA v1.18.66.3 |
| Data analysis | MEA v1.0, Trimmomatic v0.32, Bismark v0.16.3, Bowtie2 v2.2.3, STAR v2.4.0i, Cufflinks v2.1.1, Bedtools v2.22.1, Bedops v2.4.26, Samtools v1.1, Picard-tools v1.92, VisRseq v0.9.12 & v0.9.37, ChromHMM v1.12, Morpheus, DESeq2 v1.26.0, HOMER findMotifs.pl v4.11.1, MEME suite v5.1.1, Stringtie v1.3.5. |

For manuscripts utilizing custom algorithms or software that are central to the research but not yet described in published literature, software must be made available to editors/reviewers. We strongly encourage code deposition in a community repository (e.g. GitHub). See the Nature Research guidelines for submitting code & software for further information.

## Data

Policy information about availability of data

All manuscripts must include a data availability statement. This statement should provide the following information, where applicable:

- Accession codes, unique identifiers, or web links for publicly available datasets
- A list of figures that have associated raw data
- A description of any restrictions on data availability

Sequencing datasets generated in this study have been deposited in GEO under the accession number GSE141877 [https://www.ncbi.nlm.nih.gov/geo/ query/ acc.cgi?acc=GSE141877]. See Supplementary Table 2 for the full list of data analyzed for this study.

# Field-specific reporting

Please select the one below that is the best fit for your research. If you are not sure, read the appropriate sections before making your selection.

# Life sciences study design

All studies must disclose on these points even when the disclosure is negative.

| | |
|---|---|
| Sample size | No statistical method was used to pre-determine sample size. Practical considerations limited the number of samples that we could analyze. |
| Data exclusions | High-thoughput sequencing data: PCR duplicate reads or reads aligning to multiple genomic regions were excluded from analysis. This is standard practice for high-throughput sequencing datasets. |
| Replication | All datasets were generated in biological duplicates and high correlation was observed.<br>Similarly, replicate datasets from publicly available data were confirmed for high correlation and combined for analysis and visualization.<br>In addition, high reproducibility between publicly available dataset and relevant datasets generated in this study was observed. |
| Randomization | The experiments were not randomized. This study does not involve randomized samples. |
| Blinding | The investigators were not blinded to group allocation during sample collection or analysis. None of the analyses applied were based on subjective observations. |

# Reporting for specific materials, systems and methods

We require information from authors about some types of materials, experimental systems and methods used in many studies. Here, indicate whether each material, system or method listed is relevant to your study. If you are not sure if a list item applies to your research, read the appropriate section before selecting a response.

## Materials & experimental systems

| n/a | Involved in the study | |
|---|---|---|
| ☐ | ☒ | Antibodies |
| ☒ | ☐ | Eukaryotic cell lines |
| ☒ | ☐ | Palaeontology |
| ☐ | ☒ | Animals and other organisms |
| ☒ | ☐ | Human research participants |
| ☒ | ☐ | Clinical data |

## Methods

| n/a | Involved in the study | |
|---|---|---|
| ☒ | ☐ | ChIP-seq |
| ☒ | ☐ | Flow cytometry |
| ☒ | ☐ | MRI-based neuroimaging |

## Antibodies

| | |
|---|---|
| Antibodies used | DNMT3A immunofluorescence: IMGENEX #IMG-268 (0.5 μg/ml)<br>ICM immunodissection: anti-mouse IgG (Cedarlane, catalogue #CLA3340, 1:100 dilution) and guinea pig complement (Rockland, catalogue #C200-0005, 1:50 dilution) |
| Validation | As previously described: Hirasawa 2008, Genes & Development; Solter & Knowles 1975 PNAS |

## Animals and other organisms

Policy information about studies involving animals; ARRIVE guidelines recommended for reporting animal research

| | |
|---|---|
| Laboratory animals | All animal experiments were performed under the ethical guidelines of Kyushu University and Tokyo University of Agriculture.<br>All mice were at least 10-week-old at the time of each experiment. All mice were maintained at 22℃ ambient temperature, 55% humidity and 12 hour/12 hour dark/light cycle.<br>Dnmt3a matKO F1 hybrid embryos: Dnmt3a KO oocytes were generated using Dnmt3a2lox and Zp3-cre C57BL/6J mice as described previously (Hirasawa 2008, Genes & Development). Superovulation was induced using PMSG/hCG (7.5 U) and MII oocytes were collected from oviducts. Dnmt3a2lox;Zp3-cre MII oocytes were artificially inseminated with DBA/2J spermatozoa.<br>DNMT3A immunofluorescence: wild-type C57BL/6J females were mated with JF1 males and IF was performed on one-cell zygotes, as described previously (Hisarawa 2008, Genes & Development).<br>Diploid androgenones were prepared from B6D2F1/Jcl oocytes and C57BL/6NJcl spermatozoa. |
| Wild animals | Study did not involve wild animals. |
| Field-collected samples | Study did not involve samples collected from the field. |
| Ethics oversight | All animal experiments were performed under the ethical guidelines of Kyushu University and Tokyo University of Agriculture. |

Note that full information on the approval of the study protocol must also be provided in the manuscript.
